# Serum MicroRNA Profiling and Bioinformatics of Patients with Spleen-Deficiency Syndrome

**DOI:** 10.1155/2016/8726720

**Published:** 2016-11-23

**Authors:** Ze-Min Yang, Long-Hui Chen, Min Hong, Ying-Yu Chen, Xiao-Rong Yang, Si-Meng Tang, Qian-Fa Yuan, Zhen-Yu He, Wei-Wen Chen

**Affiliations:** ^1^School of Basic Courses, Guangdong Pharmaceutical University, No. 280 Waihuan Road East, Higher Education Mega Center, Panyu District, Guangzhou 510006, China; ^2^Pi-Wei Institute, Guangzhou University of Chinese Medicine, No. 12 Jichang Road, Baiyun District, Guangzhou 510405, China; ^3^Department of Traditional Chinese Medicine, First Affiliated Hospital of Guangdong Pharmaceutical University, No. 19 Nonglinxia Road, Guangzhou 510080, China; ^4^Clinical Laboratory, First Affiliated Hospital of Guangdong Pharmaceutical University, No. 19 Nonglinxia Road, Guangzhou 510080, China

## Abstract

To investigate serum microRNA (miRNA) profile and bioinformatics of patients with spleen-deficiency syndrome (SDS) and explore pathogenesis of SDS patients from miRNA levels, 10 patients with type 2 diabetes mellitus (T2DM), within which 5 patients were with SDS and the remaining were with blood stasis syndrome (BSS), and 5 healthy volunteers were recruited. Serum miRNA profiles of SDS patients were identified by quantitative PCR array. Target prediction and functional annotation for miRNAs were performed by miRSystem database. The present study identified 11 candidate serum miRNAs for SDS patients, and their targets were significantly enriched in 18 KEGG pathways and 7 GO molecular functions. Those enriched KEGG pathways included (1) metabolisms of carbohydrate, protein, amino acid, and fatty acid, (2) signaling pathways of insulin, ErbB, chemokine, calcium, and type II diabetes mellitus, (3) invasions of bacterium,* Escherichia coli*, and* Shigella* (Shigellosis), and (4) endocytosis and phagocytosis. Those enriched GO molecular functions were mainly involved in transcription regulation and regulation of metabolism. Our findings might elucidate the pathogenesis of SDS patients with disorders of substance metabolism and hypoimmunity from miRNA levels, as well as providing some miRNA biomarkers for clinical syndrome differentiation of SDS.

## 1. Introduction

Traditional Chinese Medicine (TCM) theory considers that spleen, also named as “Pi” in Chinese, is a comprehensive functional unit which is mainly involved in the digestive system as well as a wide variety of systems such as immune system. Spleen-deficiency syndrome (SDS) is one of the commonly encountered TCM syndromes and is closely associated with hypoimmunity and disorders of substance metabolism including nutrient digestion and absorption. Our previous studies in gene expression profile found that differentially expressed genes of SDS patients were mainly related to substance metabolism and immune regulation, and most of these genes were downregulated [1–3]. This finding had also been partially confirmed by other studies in cooperative institution [4–7]. These studies provided a new perspective for elucidating the physiological function of spleen and pathogenesis of SDS. Gene expression profiling enables researchers to observe expressions for tens of thousands of genes in a tissue simultaneously, which may be similar to the “holistic view” of TCM. However, it also has certain limitations. For example, gene expression profile from a single tissue is difficult to reflect the holistic status of multisystem disease of SDS, because of tissue specificity of gene expression. Furthermore, the differentially expressed gene is difficult to be used as biomarker for clinical diagnosis due to too many quantities.

MicroRNA (miRNA) is endogenously expressed, small single-stranded noncoding RNA molecules of 21–23 nucleotides, which regulate approximately 50% of all mammalian protein-coding genes and participate in many biological processes of cell development, differentiation, metabolism, immunity, and proliferation [8]. Serum or plasma miRNA is stable and is derived from different tissues or organs [9, 10]. Importantly, the specific serum miRNA expression profile constitutes the fingerprint of a physiological or disease condition [9]. A direct evidence from rat models showed that miRNA expression profiles in different tissues (pancreas, liver, adipose, and skeletal muscle) shared high similarity with that in blood samples [11]. Thus, serum miRNA expression profile could well reflect the holistic status of SDS patients and help to achieve comprehensive understanding of spleen essence. Moreover, serum miRNAs had been widely used in the study of biomarker and pathophysiological mechanism in different diseases, especially multisystem diseases including type 2 diabetes mellitus (T2DM) [10, 12]. However, few studies were performed in serum miRNA profiling of patients with SDS which had been recognized by TCM theory as one of the primary causes leading to the occurrence and development of T2DM.

The present study investigated serum miRNA profiling and bioinformatics of SDS patients by Exiqon serum/plasma miRNA qPCR (quantitative polymerase chain reaction) array, panel I, which is designed based on the miRNA screening results of thousands of patients with different diseases, and contains most of serum miRNAs of detectable, highly concerned and deeply studied by academic community. In addition, according to the method of combination of disease and syndrome, T2DM-BSS (Blood Stasis Syndrome) patients and HV (healthy volunteers) were selected as study control of T2DM-SDS patients to eliminate the effects of T2DM and syndrome types of TCM and to obtain the candidate serum miRNAs for SDS patients. The aim of the present study was to investigate the substance basis of miRNA and pathogenesis of SDS patients from the holistic level. It might provide further evidences for study on the scientific essence of spleen and clinical syndrome differentiation of SDS.

## 2. Materials and Methods

### 2.1. Ethics Statement

The present study was approved by the Ethics Committee of the First Affiliated Hospital of Guangzhou University of Chinese Medicine. All participants signed written informed consent.

### 2.2. Diagnostic Standard of T2DM

Diagnostic standard of T2DM was set up by the American Diabetes Association (ADA) as follows [13]: (1) a fasting plasma glucose (FPG) level of 126 mg/dL (7.0 mmol/L) or higher, (2) a 2-hour plasma glucose level of 200 mg/dL (11.1 mmol/L) or higher during a 75-g oral glucose tolerance test (OGTT), or (3) a random plasma glucose of 200 mg/dL (11.1 mmol/L) or higher in a patient with classic symptoms of hyperglycemia or hyperglycemic crisis.

### 2.3. Diagnostic Standard of Syndrome Differentiation

The diagnostic standard of syndrome differentiation was set up according to “Guiding Principle of Clinical Research on New Drugs of Chinese Medicine” and the clinical experience of authors' institute [1, 14, 15]. Syndrome differentiation was performed by two associate chief physicians of Chinese Medicine.

The diagnostic criteria for SDS were described below. The dominant symptom items were (1) pale tongue proper with thin-white coating, (2) poor appetite, (3) abdominal distension, and (4) loose stool or diarrhea. The secondary items were (1) emaciated, (2) feebleness, and (3) weak pulse. The dominant item (1) was necessary for the diagnosis of SDS. To make the diagnosis, it also required two more dominant items or one dominant symptom plus at least two secondary symptoms.

The diagnostic criteria for BSS were as follows. The dominant symptom items were (1) localized pain, prickling, or nonpalpable abdominal pain, (2) purple-colored tongue body or tongue body with petechial, sublingual vein varicosity, (3) pathological lumps including gut swelling, neoplasm, inflammatory or noninflammatory masses, or tissue proliferation, (4) vascular ecchymosis, including cyanotic lip, gingival or acra, red thread in skin surface, or abdominal veins varicosity, (5) bleeding and melena, petechial, or bloody ascites, (6) menstrual disorders including dysmenorrhea, black or clotted menstruation, or hypogastric pain, and (7) uneven or intermittent pulse or acrotism. The secondary items were (1) scaly, dry skin, (2) limb torpidity or hemiplegia, (3) insanity, mania, amnesia, or topoparesthesia, and (4) a history of trauma, surgery, and induced abortion. The diagnosis of BSS was able to be confirmed when a patient had more than 2 dominant symptom items or 1 dominant symptom item plus at least 2 secondary symptom items.

### 2.4. Inclusion and Exclusion Criteria for T2DM Patients with SDS or BSS

Inclusion criteria were as follows: patients (1) were clinically diagnosed as T2DM, (2) were of the Chinese medicine syndrome type differentiated as SDS or BSS, (3) were of the age between 48 and 70 years, and (4) signed the informed consent form. Exclusion criteria were as follows: patients (1) were with other endocrine diseases in addition to T2DM, or combined with severe complications due to T2DM, (2) were with infectious or inflammatory diseases, (3) were with psychiatric or serious somatic diseases, (4) were with dyslipidemia, or (5) were in pregnancy or lactation stage.

### 2.5. Judgment Standard and Enrollment of Healthy Volunteers

The healthy volunteers (HV) aged between 48 and 70 years were screened by chief physician (Min Hong) through interview and physical examination. They were free of endocrine diseases including T2DM, infectious and inflammatory diseases, psychiatric and serious somatic diseases, and dyslipidemia. They should have no apparent TCM syndromes, with normal figure of tongue and pulse. They were also excluded if they were overweight or obese, presented a family history of diabetes, or long-term medication use, or failed to sign the informed consent.

### 2.6. Grouping

Ten patients of T2DM were recruited from the First Affiliated Hospital of Guangzhou University of Chinese Medicine during October 2013 to December 2013 and were divided into two groups according to the TCM syndrome type. Five patients (3 females and 2 males) aged from 48 to 65 (57.0 ± 7.0) years were assigned to the SDS group, while the other 5 patients (3 females and 2 males) aged from 49 to 70 (59.4 ± 9.0) years were assigned to the BSS group. Five healthy volunteers (3 females and 2 males), assigned as HV group, were also recruited from the Physical Examination Center, the First Affiliated Hospital of Guangdong Pharmaceutical University during December 2013 to April 2014. The average age of all participants was 56.4 ± 3.7 (from 51 to 61) years. No significant differences in age and sex were observed among the three study groups (*P* > 0.05).

### 2.7. Serum Sample Collection of Participants

More than 3 mL whole venous bloods were collected into a vacuum tube sans anticoagulants in a fasting state between 7:00 am and 9:00 am. After completing blood coagulation in 4°C refrigerator for 1 hour, samples were centrifuged at 1,000 rpm for 10 min at 4°C to obtain serum. Each 500 *μ*L of serum was then packed in a frozen RNase-free storage tube and then stored at −80°C until use. The whole procedure was completed within 2 hours after blood sampling.

### 2.8. RNA Isolation

Total RNAs, including miRNA, were isolated from the serum using Trizol reagent (Invitrogen Life Technologies, USA) according to the manufacturer's protocol. RNA concentration and purity were determined by NanoDrop ND-1000 spectrophotometer (Thermo Fisher Scientific, USA). RNA samples that met *A*(absorbance)_260_/*A*_280_ value >1.7 and RNA concentration >60 *μ*g/*μ*L were used for the miRNA real-time qPCR array.

### 2.9. miRNA Real-Time qPCR Array

The miRCURY LNA™ universal RT microRNA PCR array, serum/plasma panel I (Exiqon, Danmark), containing 372 human mature miRNAs, was selected to detect serum miRNA expression profiles. Approximately 20–25 ng of total RNAs containing miRNA were reverse-transcribed into cDNA using the MicroRNA Reverse Transcription Kit and RT Primer Pools (Exiqon, Denmark). Then, cDNA was served as template for miRNA real-time qPCR in ABI PRISM7900 system (Applied Biosystems, USA). Thermal cycling was denatured at 95°C for 10 min, followed by 38 cycles of 95°C for 10 s and 60°C for 60 s. Melting curve was performed at the end of the PCR cycle. All procedures were taken according to the manufacturer's protocol.

### 2.10. Determination of Differentially Expressed miRNA

The threshold cycle (CT) values of each sample were determined by the number of PCR cycle and threshold value. Undetectable data was assigned a default CT value of 38. The CT values of individual miRNA were normalized to the housekeeping gene (SNORD38B), which had stable CT value in all samples. The miRNA expression levels for each sample were calculated using 2^−ΔCT^ (ΔCT = CT of target miRNA − CT of SNORD38B). Differences in miRNA expression between experimental (SDS groups) and control groups (HV or BSS group) were compared using two-tailed Student's *t*-test. The relative expression levels (fold changes) of miRNAs between the two groups were calculated using 2^−(ΔCT  of  experimental  group − ΔCT  of  control  group)^. The miRNAs that matched *P* < 0.05 and fold change >2.0 or <0.5 were defined as differentially expressed between the two groups. Data was expressed as mean ± SD. The data was analyzed with the GenEx qPCR (Exiqon, Denmark) and SPSS 18.0 (IBM, USA) software. To determine the candidate miRNAs of SDS patients, the VENNY tool [16] was used to compare differentially expressed miRNAs between SDS and HV groups with that of between SDS and BSS groups via Venn diagrams.

### 2.11. Target Prediction of miRNA and Functional Annotation of the Predicted Target

To evaluate functions of candidate miRNAs of SDS patients, target prediction and functional annotation were performed using the miRSystem database (version 20150312) [17], which is an integrated system for characterizing enriched functions and pathways of miRNA targets. In target prediction, the miRSystem database integrates 2 experimentally validated databases, TarBase (version 7.0) and miRecords (November 2010 release), and 7 target gene prediction algorithms including Diana-microT (version 4.0), miRanda (August 2010 release), miRBridge (April 2010 release), PicTar (March 2007 release), PITA (August 2008 release), RNA22 (version 2.0), and Targetscan (version 6.0). In functional annotation, miRSystem database integrates 5 databases including KEGG (Kyoto Encyclopedia of Genes and Genomes), Biocarta, Pathway Interaction Database (human only), Reactome (human only), and GO (Gene Ontology) molecular function. To identify significantly enriched pathways or biological function, several statistical approaches, including O/E (observed to expected) ratios of gene targets, hypergeometric and empirical *P*-value from permutation, and pathway ranking score from miRNA relative expression value (fold change), were also provided in miRSystem database [17]. In the present study, miRNAs and their fold changes were submitted to miRSystem database. Only validated targets or miRNA-target interactions identified by at least 4 prediction programs were considered for annotation of KEGG pathway and GO molecular function. Moreover, parameter settings were 25–500 genes in biological functions/pathways, O/E ratios ≥1.8, and ranking score ≥2.0.

## 3. Results

### 3.1. Serum miRNA Profile of SDS Patients

**Figure 1:**
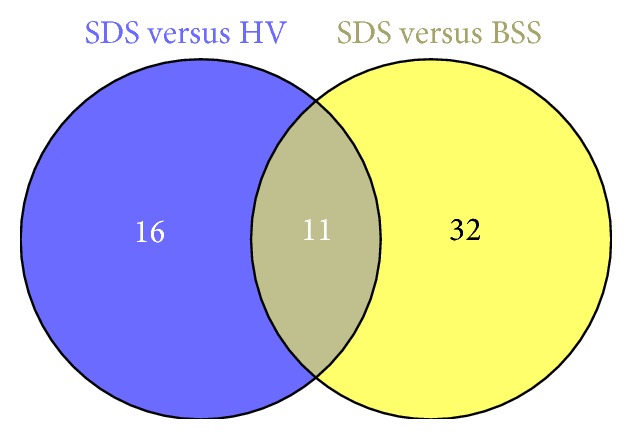
Venn diagrams of differential expression miRNAs between T2DM-SDS patients and T2DM-BSS patients or HV.

Quantitative PCR arrays were introduced to compare the serum miRNA expression profiles between T2DM-SDS patients and healthy volunteers or T2DM-BSS patients. Out of 372 tested mature miRNAs, 27 miRNAs were significant differentially expressed between T2DM-SDS patients and healthy volunteers, with 5 downregulated and 22 upregulated miRNAs. Forty-three miRNAs were significant differentially expressed between patients of T2DM-SDS and T2DM-BSS, with 1 downregulated and 42 upregulated miRNAs (Figure 1). Among all these differentially expressed miRNAs, 11 upregulated miRNAs were shared by both, which might be candidate serum miRNAs for SDS patients (Figure 1 and Table 1).

### 3.2. miRNA Target Prediction and Functional Annotation

The predicted targets of the 11 candidate serum miRNAs of SDS patients were identified by miRSystem database. Results indicated that 2,713 putative targets were regulated by these miRNAs. Among these targets, O/E ratios of 137 targets were more than 1.8. Function annotation showed that these 137 targets were significantly enriched in 18 KEGG pathways (Table 2) and 7 GO molecular functions (Table 3). These KEGG pathways were involved in (1) metabolism of nutrients, including carbohydrate, protein, amino acid, and fatty acid, (2) signaling pathway, including signaling of insulin, ErbB, chemokine, calcium, and type II diabetes mellitus, (3) invasion of pathogenic factors, including bacterium,* Escherichia coli*, and* Shigella* (Shigellosis), and (4) endocytosis and phagocytosis. These GO molecular functions were involved in (1) regulation of transcription, including transcription factor activity and chromatin binding, and (2) regulation of metabolism, including bindings of carboxylic acid and lipid, and enzyme activator activity. These miRNAs and their targets participated in all enriched KEGG pathways and GO molecular functions were shown in Tables S1 and S2 from Supplementary Material available online at http://dx.doi.org/10.1155/2016/8726720.

**Figure 2:**
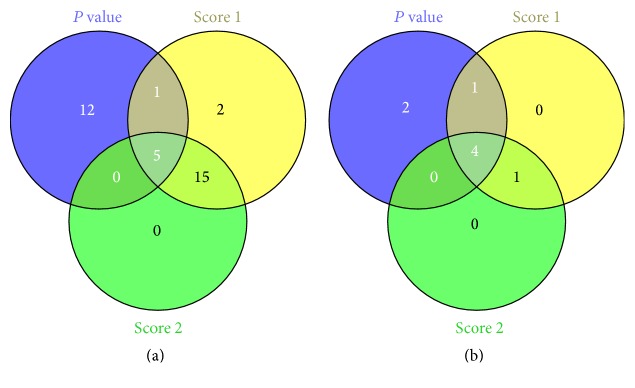
Venn diagrams of KEGG pathway and GO molecular function annotations based on *P* and score value. Score 1 indicated number of enriched biological functions in ranking score >2.0 from T2DM-SDS patients versus HV. Score 2 indicated number of enriched biological functions in ranking score >2.0 from T2DM-SDS patients versus T2DM-BSS patients. (a) denoted KEGG pathway and (b) denoted GO molecular function.

Subsequently, 11 candidate miRNAs of SDS patients and their fold changes were incorporated into an additional weighted pathway-ranking method to identify the enriched biological functions. Results in KEGG pathway indicated that 23 pathways matched ranking score ≥2.0 were identified in T2DM-SDS patients versus HV, and 20 pathways matched ranking score ≥2.0 were identified in T2DM-SDS patients versus T2DM-BSS patients (shown in Tables S3 and S4 from Supplementary Material). Furthermore, 5 pathways were shared by both when *P* value was imported (Figure 2(a)). The 5 top canonical KEGG pathways were ErbB signaling pathway, insulin signaling pathway, calcium signaling pathway, endocytosis, and bacterial invasion of epithelial cells, and their scores were reduced in turn. The miRNAs and predicted targets in the 5 top canonical KEGG pathways were shown in Table 4. Results in GO molecular function indicated that 6 functions matched ranking score ≥2.0 were identified in T2DM-SDS patients versus HV, and 5 functions matched ranking score ≥2.0 were identified in T2DM-SDS patients versus T2DM-BSS patients (shown in Tables S5 and S6 from Supplementary Material). Moreover, 4 pathways were shared by both when *P* value was imported (Figure 2(b)). The 4 top canonical GO molecular functions were transcription factor binding transcription factor activity, transcription repressor activity, RNA polymerase II transcription factor activity, and chromatin binding, and their scores were declined in turn. The miRNAs and targets in the 4 top canonical GO molecular functions were shown in Table 5.

## 4. Discussion

For the first time, the present study revealed differential serum miRNA expression profile between T2DM-SDS patients and T2DM-BSS patients or healthy volunteers by low density miRNA qPCR array. We found 11 candidate serum miRNAs of SDS patients. Target prediction and functional annotation by miRSystem database showed that targets of these miRNAs were significantly enriched in several KEGG pathways including nutrient metabolism, signaling pathway, bacterial invasion, and endocytosis and in some GO molecular functions including transcription and metabolism regulation. Our findings provided further evidences for study on the pathogenesis of SDS patients and for clinical syndrome differentiation of SDS.

TCM theory considers that the main physiological function of spleen is to govern transportation and transformation of substances. For the transportation function, spleen is responsible for digestion, absorption, and transportation of cereal essence. For the transformation function, spleen transforms cereal essence to essence, Qi (it is the basic term of TCM and related to energy), blood and body fluid in order to nourish the body. Thus spleen is closely related to the metabolism and transportation of substance including nutrients and signal transduction in Western Medicine. SDS is a TCM syndrome, which is manifested as deficiency of spleen-Qi and dysfunction of spleen governing transportation and transformation. A large number of research results in enzymology and digestive system after 1980s [18], and a few results in gene expression profiles after 2000s [1, 2, 5, 6], had confirmed that SDS patients had the disorder of digestion and absorption and the abnormal metabolism of substances. Nonetheless, the understanding of the pathogenesis of SDS was not comprehensive because of particularity of SDS disease itself and shortages of these research methods. Spleen was a comprehensive functional unit and its anatomical location is not clear; thus pathogenesis of SDS was complex and complicated. One or several indicators were difficult to fully reflect the pathogenesis of SDS. Moreover, gene expression profiling was not suitable for study of multisystem diseases. However, serum miRNA expression profile, as a novel method, could overcome these shortcomings as mentioned in the introduction. Moreover, serum miRNA expression profile in SDS patients had not been reported.

In the present study, for the first time, we reported 11 serum miRNAs from SDS patients and all these miRNAs were upregulated. Among these 11 miRNAs, miR-9, miR-96, miR-153, and miR-200a had been confirmed to be related to the occurrence of type 2 diabetes [10, 12, 19]. Furthermore, miR-9, miR-96, and miR-153 regulated insulin secretory [12, 19], while miR-200a and miR-96 regulated insulin receptor pathway [12]. Besides, miR-124-3p controlled glucagon release by directly targeting iGluR2 and iGluR3 [20]; miR-137 regulated proliferation and differentiation of human adipose tissue stromal cells by targeting CDC42 [21]; miR-135a-5p and miR-135b-5p controlled adipogenesis and atherosclerosis, respectively [22, 23]; miR-141-3p contributed to mitochondrial dysfunction in high-fat-diet induced obesity by inhibiting PTEN [24]. These functional studies in miRNAs related to SDS patients showed that there were abnormal metabolism of glucose and lipid in SDS patients from the macroscopic view. In addition, functional annotation in the present study showed that targets downregulated by 11 miRNAs of SDS patients were significantly enriched in several KEGG pathways related to nutrient metabolism, signaling pathway, and endocytosis and in some GO molecular functions including regulation of transcription and metabolism. These findings further indicated SDS patients had dysfunction of nutrient metabolism and transportation and signal transduction. Duan et al. found that SDS model rats had lower expressions of key factors from Ca^2+^/CaMK II signaling pathways in skeletal muscle than control rats [25]. In our previous studies in chronic superficial gastritis patients (CSG) with SDS [1, 2], we found that more than 70% differentially expressed genes between CGS-SDS patients and CGS-SSHS (spleen-stomach hygropyrexia syndrome) patients or healthy volunteers were associated with metabolism of nutrients including carbohydrate, protein, amino acid, lipid and nucleic acid, and metal ion metabolism. Furthermore, genes related to carbohydrate metabolisms participated in glycogen and glycan metabolic processes; genes related to protein metabolism participated in signaling pathways, amino acid metabolism, and protein ubiquitination and targeting transport including endocytosis; genes related to nucleic acid metabolism participated in regulations of replication and transcription; genes related to metal ion metabolism were mainly calcium binding protein. Interestingly, among all these genes involved in metabolism, more than 80% genes were downregulated in GSG-SDS patients. Moreover, downregulated ACADVL (acyl-CoA dehydrogenase, very long chain) and ACAA2 (acetyl-coenzyme A acyltransferase 2) [1, 2, 5], which catalyze the first and last steps of the mitochondrial fatty acid beta-oxidation pathway, were targeted by upregulated 135a-5p and miR-124-3p in current study, respectively. Thus, our previous reports of gene expression profiles from CSG-SDS patients strongly supported the current research results. They elucidated scientific connotation of spleen governing transportation and transformation and pathogenesis of SDS patients with disorders of substance metabolism from gene and miRNA levels, respectively. In addition, in the present study, we found that all the KEGG pathways and GO molecular functions related to nutrient metabolism were deleted from the significantly enriched biological functions when weighted pathway-ranking score ≥2.0 was used to identify the enriched biological functions. These findings might illustrate that spleen governing transportation was more important than spleen governing transformation in physiological function of spleen.

TCM theory considers that spleen is the foundation of acquired constitution and that the reinforced function of spleen is responsible for individual's insusceptibility to pathogenic factors in the four seasons. Hence, spleen can protect the body from external pathogenic factor, and thus spleen-deficiency would break out all kinds of diseases and ailments. Accumulating clinical observations has indicated that SDS patients usually manifest weak constitution and hypoimmunity, as well as dysfunction of the digestive system. Luo et al. found that the pathogenesis of SDS patients had genomic basis associated with immune, and SDS patients had disorder of the immune functions [7]. In the present study, we found that targets downregulated by the 11 miRNAs of SDS patients were significantly enriched in 3 KEGG pathways related to invasion of pathogenic factor, including bacterium,* Escherichia coli*, and* Shigella* (Shigellosis). Furthermore, KEGG pathway of bacterial invasion of epithelial cells was one of the 5 top canonical KEGG pathways. These findings strongly indicated that SDS patients had a reduced resistance to external pathogenic factor. In our recent studies about gene expression profile from peripheral white blood cells of T2DM-SDS patients [3, 15], we found that approximate 30% of differentially expressed genes between T2DM-SDS patients and T2DM-BSS patients or healthy volunteers were associated with immune regulation or response. Furthermore, more than 60% genes were downregulated in all these differentially expressed genes. Our previous studies of gene expression profiles from T2DM-SDS patients supported the current study. In addition, among 11 miRNAs of SDS patients in the present study, miR-219-5p was confirmed to directly regulate inflammatory adipokine expression and macrophage innate immune response [26]. Hence, all these results elucidated scientific connotation of spleen governing protection of the body from external pathogenic factor and pathogenesis of SDS patients with hypoimmunity from gene and miRNA levels, respectively.

Although the present study obtained some meaningful results in the scientific essence of spleen and pathogenesis of SDS patients, there were some possible limitations. For example, the present sample size was limited, and verifications of predicted targets were not performed. Thus further researches were needed in the future.

## Supplementary Material

Table S1: miRNAs and their targets in enriched KEGG pathways; Table S2: miRNAs and their targets in enriched GO molecular functions; Table S3: KEGG Pathway Ranking Summary (PDS vs HV, Score>2.0); Table S4: KEGG Pathway Ranking Summary (PDS vs BSS, Score>2.0); Table S5: GO Molecular function Ranking Summary (PDS vs HV, Score>2.0); Table S6: GO Molecular function Ranking Summary (PDS vs BSS, Score>2.0).

## Figures and Tables

**Table 1:** Eleven candidate serum miRNAs of SDS patients.

miRNAs	SDS versus HV		SDS versus BSS	
	Fold change	*P* value	Fold change	*P* value
miR-153	7.14 ± 0.48	0.0096	8.65 ± 0.48	0.0091
miR-96-5p	5.04 ± 0.48	0.0038	2.60 ± 0.48	0.0463
miR-9-5p	5.00 ± 0.36	0.0197	6.35 ± 0.36	0.0075
miR-137	4.80 ± 0.41	0.0121	10.67 ± 0.41	0.0032
miR-135a-5p	4.67 ± 0.59	0.0085	3.67 ± 0.69	0.0052
miR-124-3p	3.64 ± 0.46	0.0091	5.25 ± 0.46	0.0097
miR-135b-5p	3.43 ± 0.36	0.0498	5.50 ± 0.36	0.0193
miR-219-5p	3.06 ± 0.62	0.0031	4.47 ± 0.62	0.0124
miR-136-5p	2.80 ± 0.56	0.0069	6.74 ± 0.56	0.0069
miR-141-3p	2.66 ± 0.55	0.0246	3.23 ± 0.55	0.0350
miR-200a-3p	2.03 ± 0.63	0.0308	2.62 ± 0.63	0.0370

**Table 2:** KEGG pathways from 11 candidate serum miRNAs of SDS patients.

Term	Term ID	Targets in the term	miRNAs in the term	*P* value
Insulin signaling pathway	hsa04910	4	8	0.0090
ErbB signaling pathway	hsa04012	3	7	0.0150
Chemokine signaling pathway	hsa04062	4	7	0.0240
Endocytosis	hsa04144	4	6	0.0286
Calcium signaling pathway	hsa04020	4	5	0.0198
Carbohydrate digestion and absorption	hsa04973	2	5	0.0270
Ubiquitin mediated proteolysis	hsa04120	3	5	0.0421
Bacterial invasion of epithelial cells	hsa05100	4	4	0.0009
Fc*γ*R-mediated phagocytosis	hsa04666	3	4	0.0181
Arginine and proline metabolism	hsa00330	2	4	0.0400
Galactose metabolism	hsa00052	2	3	0.0108
Amino sugar and nucleotide sugar metabolism	hsa00520	2	3	0.0315
Starch and sucrose metabolism	hsa00500	2	3	0.0387
Pathogenic *Escherichia coli* infection	hsa05130	3	2	0.0047
Valine, leucine, and isoleucine degradation	hsa00280	2	2	0.0281
Type II diabetes mellitus	hsa04930	2	2	0.0315
Shigellosis	hsa05131	2	2	0.0489
Fatty acid metabolism	hsa00071	3	1	0.0023

**Table 3:** GO molecular functions from 11 candidate serum miRNAs of SDS patients.

Term	Term ID	Targets in the term	miRNAs in the term	*P* value
Transcription repressor activity	GO:0016564	6	9	0.0069
Enzyme activator activity	GO:0008047	6	9	0.0148
Lipid binding	GO:0008289	6	9	0.0222
Transcription factor binding Transcription factor activity	GO:0000989	7	7	0.0061
Carboxylic acid binding	GO:0031406	4	5	0.0024
Chromatin binding	GO:0003682	4	5	0.0240
RNA polymerase II transcription factor activity	GO:0003702	5	4	0.0186

**Table 4:** miRNAs and their targets in 5 top canonical KEGG pathways (score down).

Term	Targets in the term	miRNAs in the term
ErbB signaling pathway	ELK1: ELK1, member of ETS oncogene family	miR-135a-5p,miR-135b-5p,miR-136-5p,miR-219a-5p
	SHC1: SHC (Src homology 2 domain containing) transforming protein 1	miR-124-3p,miR-219a-5p,miR-9-5p,miR-96-5p
	SHC3: SHC (Src homology 2 domain containing) transforming protein 3	miR-124-3p
Insulin signaling pathway	ELK1	As above
	SHC1	As above
	HK3: hexokinase 3 (white cell)	miR-137
	SHC3	As above
Calcium signaling pathway	GNAQ: guanine nucleotide binding protein (G protein), q polypeptide	miR-135a-5p,miR-135b-5p,miR-96-5p
	SLC25A5: solute carrier family 25 (mitochondrial carrier; adenine nucleotide translocator), member 5	miR-135a-5p,miR-135b-5p,miR-137
	CACNA1G: calcium channel, voltage-dependent, T type, alpha 1G subunit	miR-137,miR-96-5p
	SPHK2: sphingosine kinase 2	miR-137,miR-153-3p
Endocytosis	GRK6:G protein-coupled receptor kinase 6	miR-137,miR-141-3p,miR-200a-3p
	EPN1: epsin 1	miR-141-3p,miR-200a-3p
	STAMBP: STAM binding protein	miR-135a-5p,miR-135b-5p
	CHMP4A: charged multivesicular body protein 4A	miR-96-5p
Bacterial invasion of epithelial cells	SHC1	As above
	ARPC1A: actin related protein 2/3 complex, subunit 1A, 41 kDa	miR-9-5p
	ARPC1B: actin related protein 2/3 complex, subunit 1B, 41 kDa	miR-124-3p
	SHC3	As above

**Table 5:** miRNAs and their targets in 4 top canonical GO molecular functions (score down).

Term	Targets in the term	miRNAs in the term
Transcription factor binding transcription factor activity	TAF12: TAF12 RNA polymerase II, TATA box binding protein (TBP) associated factor, 20 kDa	miR-137,miR-141-3p,miR-200a-3p
	CSDA	miR-137,miR-9-5p
	DYRK1B: dual-specificity tyrosine-(Y)-phosphorylation regulated kinase 1B	miR-135a-5p,miR-135b-5p,miR-9-5p
	PIAS2: protein inhibitor of activated STAT, 2	miR-137
	DDX5: DEAD (Asp-Glu-Ala-Asp) box helicase 5	miR-200a-3p,miR-141-3p
	SIRT1: sirtuin 1	miR-124-3p,miR-135a-5p,miR-135b-5p,miR-141-3p,miR-200a-3p,miR-96-5p
	ELK3: ELK3, ETS-domain protein (SRF accessory protein 2)	miR-124-3p,miR-135a-5p,miR-135b-5p
Transcription repressor activity	TWIST2: twist family bHLH transcription factor 2	miR-124-3p
	REST: RE1-silencing transcription factor	miR-9-5p
	NKAP: NFKB activating protein	miR-124-3p
	YBX1: Y box binding protein 1	miR-153-3p,miR-137
	KLF4: Kruppel-like factor 4 (gut)	miR-124-3p,miR-135a-5p,miR-135b-5p,miR-219a-5p
	SIRT1	As above
RNA polymerase II transcription factor activity	TAF12	As above
	CSDA	As above
	HTATSF1: HIV-1 Tat specific factor 1	miR-141-3p
	MNX1: motor neuron and pancreas homeobox 1	miR-200a-3p,miR-141-3p
	PIAS2	As above
Chromatin binding	REST	As above
	NKAP	As above
	ING2: inhibitor of growth family, member 2	miR-153-3p
	YBX2:Y box binding protein 2	miR-135a-5p, miR-135b-5p
