# Supplementary material for: Serum MicroRNA Profiling and Bioinformatics of Patients with Spleen-Deficiency Syndrome
Source: Evid Based Complement Alternat Med. 2016 Nov 23;2016:8726720. doi: 10.1155/2016/8726720 (PMC5141567; doi:10.1155/2016/8726720)
Supplement: Supplementary file 1 — Table S1: miRNAs and their targets in enriched KEGG pathways; Table S2: miRNAs and their targets in enriched GO molecular functions; Table S3: KEGG Pathway Ranking Summary (PDS vs HV, Score>2.0); Table S4: KEGG Pathway Ranking Summary (PDS vs BSS, Score>2.0); Table S5: GO Molecular function Ranking Summary (PDS vs HV, Score>2.0); Table S6: GO Molecular function Ranking Summary (PDS vs BSS, Score>2.0). [file 8726720.f1.docx]

**TABLE S1: miRNAs and their targets** **in enriched KEGG pathways**

| Term | Targets in the term | miRNAs in the term |
| --- | --- | --- |
| Bacterial invasion of epithelial cells | SHC1:SHC (Src homology 2 domain containing) transforming protein 1 | miR-124-3p,miR-219a-5p,miR-9-5p,miR-96-5p |
|  | ARPC1A:actin related protein 2/3 complex, subunit 1A, 41kDa | miR-9-5p |
|  | ARPC1B:actin related protein 2/3 complex, subunit 1B, 41kDa | miR-124-3p |
|  | SHC3:SHC (Src homology 2 domain containing) transforming protein 3 | miR-124-3p |
| Fatty acid metabolism | PECI | miR-124-3p |
|  | ACADVL:acyl-CoA dehydrogenase, very long chain | miR-124-3p |
|  | ACAA2:acetyl-CoA acyltransferase 2 | miR-124-3p |
| Pathogenic *Escherichia coli* infection | ARPC1A | as above |
|  | ARPC1B | as above |
|  | TUBB6:tubulin, beta 6 class V | miR-124-3p |
| Insulin signaling pathway | ELK1:ELK1, member of ETS oncogene family | miR-135a-5p,miR-135b-5p,miR-136-5p,miR-219a-5p |
|  | SHC1 | as above |
|  | HK3:hexokinase 3 (white cell) | miR-137 |
|  | SHC3 | as above |
| Galactose metabolism | UGP2:UDP-glucose pyrophosphorylase 2 | miR-137,miR-141-3p,miR-200a-3p |
|  | HK3 | as above |
| ErbB signaling pathway | ELK1 | as above |
|  | SHC1 | as above |
|  | SHC3 | as above |
| FcγR-mediated phagocytosis | SPHK2:sphingosine kinase 2 | miR-153-3p,miR-137 |
|  | ARPC1A | as above |
|  | ARPC1B | as above |
| Calcium signaling pathway | GNAQ:guanine nucleotide binding protein (G protein), q polypeptide | miR-135a-5p,miR-135b-5p,miR-96-5p |
|  | SLC25A5:solute carrier family 25 (mitochondrial carrier; adenine nucleotide translocator), member 5 | miR-135a-5p,miR-135b-5p,miR-137 |
|  | CACNA1G:calcium channel, voltage-dependent, T type, alpha 1G subunit | miR-137,miR-96-5p |
|  | SPHK2 | as above |
| Chemokine signaling pathway | SHC1 | as above |
|  | GRK6:G protein-coupled receptor kinase 6 | miR-137,miR-141-3p,miR-200a-3p |
|  | GNG10:guanine nucleotide binding protein (G protein), gamma 10 | miR-124-3p |
|  | SHC3 | as above |
| Carbohydrate digestion and absorption | ATP1B1:ATPase, Na+/K+ transporting, beta 1 polypeptide | miR-135a-5p,miR-135b-5p,miR-137,miR-141-3p,miR-9-5p |
|  | HK3 | as above |
| Valine leucine and isoleucine degradation | ACAA2 | as above |
|  | AUH:AU RNA binding protein/enoyl-CoA hydratase | miR-9-5p |
| Endocytosis | GRK6 | as above |
|  | EPN1:epsin 1 | miR-141-3p,miR-200a-3p |
|  | STAMBP:STAM binding protein | miR-135a-5p,miR-135b-5p |
|  | CHMP4A:charged multivesicular body protein 4A | miR-96-5p |
| Type II diabetes mellitus | CACNA1G | as above |
|  | HK3 | as above |
| Amino sugar and nucleotide sugar metabolism | UGP2 | as above |
|  | HK3 | as above |
| Starch and sucrose metabolism | UGP2 | as above |
|  | HK3 | as above |
| Arginine and proline metabolism | NAGS:N-acetylglutamate synthase | miR-135a-5p,miR-135b-5p |
|  | P4HA2:prolyl 4-hydroxylase, alpha polypeptide II | miR-124-3p,miR-9-5p |
| Ubiquitin mediated proteolysis | SIAH1:siah E3 ubiquitin protein ligase 1 | miR-135a-5p,miR-135b-5p,miR-141-3p,miR-200a-3p |
|  | DET1:de-etiolated homolog 1 (Arabidopsis) | miR-135a-5p,miR-135b-5p |
|  | PIAS2:protein inhibitor of activated STAT, 2 | miR-137 |
| Shigellosis | ARPC1A | as above |
|  | ARPC1B | as above |

**TABLE S2: miRNAs and their targets in enriched GO molecular functions**

| Term | Targets in the term | miRNAs in the term |
| --- | --- | --- |
| Transcription repressor activity | TWIST2:twist family bHLH transcription factor 2 | miR-124-3p |
|  | REST:RE1-silencing transcription factor | miR-9-5p |
|  | NKAP:NFKB activating protein | miR-124-3p |
|  | YBX1:Y box binding protein 1 | miR-153-3p,miR-137 |
|  | KLF4:Kruppel-like factor 4 (gut) | miR-124-3p,miR-135a-5p,miR-135b-5p,miR-219a-5p |
|  | SIRT1:sirtuin 1 | miR-124-3p,miR-135a-5p,miR-135b-5p,miR-141-3p,miR-200a-3p,miR-96-5p |
| Enzyme activator activity | TBC1D19:TBC1 domain family, member 19 | miR-153-3p,miR-137 |
|  | ARHGEF1:Rho guanine nucleotide exchange factor (GEF) 1 | miR-124-3p |
|  | ALOX5AP:arachidonate 5-lipoxygenase-activating protein | as above |
|  | GNAQ | as above |
|  | DNAJC1:DnaJ (Hsp40) homolog, subfamily C, member 1 | miR-124-3p |
|  | STK3:serine/threonine kinase 3 | miR-137,miR-141-3p,miR-200a-3p,miR-9-5p |
| Lipid binding | EPN1 | as above |
|  | ANXA7:annexin A7 | miR-124-3p,miR-135a-5p,miR-135b-5p |
|  | SNX25:sorting nexin 25 | miR-137,miR-9-5p |
|  | CHMP4A | as above |
|  | YBX2:Y box binding protein 2 | miR-135a-5p,miR-135b-5p |
|  | SHC1 | as above |
| Transcription factor binding transcription factor activity | TAF12:TAF12 RNA polymerase II, TATA box binding protein (TBP)-associated factor, 20kDa | miR-137,miR-141-3p,miR-200a-3p |
|  | CSDA | miR-137,miR-9-5p |
|  | DYRK1B:dual-specificity tyrosine-(Y)-phosphorylation regulated kinase 1B | miR-135a-5p,miR-135b-5p,miR-9-5p |
|  | PIAS2 | as above |
|  | DDX5:DEAD (Asp-Glu-Ala-Asp) box helicase 5 | miR-200a-3p,miR-141-3p |
|  | SIRT1 | as above |
|  | ELK3:ELK3, ETS-domain protein (SRF accessory protein 2) | miR-124-3p,miR-135a-5p,miR-135b-5p |
| Carboxylic acid binding | PECI | as above |
|  | P4HA2 | as above |
|  | ACOT7:acyl-CoA thioesterase 7 | miR-141-3p,miR-200a-3p,miR-9-5p |
|  | ALOX5AP | as above |
| Chromatin binding | REST | as above |
|  | NKAP | as above |
|  | ING2:inhibitor of growth family, member 2 | miR-153-3p |
|  | YBX2 | as above |
| RNA polymerase II transcription factor activity | TAF12 | as above |
|  | CSDA | as above |
|  | HTATSF1:HIV-1 Tat specific factor 1 | miR-141-3p |
|  | MNX1:motor neuron and pancreas homeobox 1 | miR-200a-3p,miR-141-3p |
|  | PIAS2 | as above |

**TABLE S3: KEGG Pathway Ranking Summary (PDS vs HV, Score>2.0)**

| Term | Term ID | Total genes of the term | Union targets in the term | miRNAs in the term | Score |
| --- | --- | --- | --- | --- | --- |
| Axon guidance | 4360 | 129 | 55 | 10 | 4.473 |
| Pathways in cancer | 5200 | 325 | 105 | 11 | 4.147 |
| Erbb signaling pathway | 4012 | 87 | 42 | 11 | 3.309 |
| Focal adhesion | 4510 | 199 | 68 | 11 | 3.263 |
| Regulation of actin cytoskeleton | 4810 | 213 | 76 | 11 | 3.222 |
| Insulin signaling pathway | 4910 | 137 | 47 | 11 | 3.133 |
| TGF-beta signaling pathway | 4350 | 84 | 30 | 11 | 3.001 |
| Wnt signaling pathway | 4310 | 150 | 47 | 11 | 2.918 |
| MAPK signaling pathway | 4010 | 272 | 80 | 11 | 2.782 |
| Neurotrophin signaling pathway | 4722 | 127 | 47 | 11 | 2.694 |
| Gap junction | 4540 | 90 | 32 | 10 | 2.572 |
| Calcium signaling pathway | 4020 | 177 | 44 | 11 | 2.507 |
| Chronic myeloid leukemia | 5220 | 73 | 33 | 11 | 2.449 |
| Gnrh signaling pathway | 4912 | 101 | 32 | 11 | 2.429 |
| Prostate cancer | 5215 | 89 | 33 | 11 | 2.425 |
| Long-term potentiation | 4720 | 70 | 29 | 11 | 2.417 |
| Gastric acid secretion | 4971 | 74 | 25 | 11 | 2.261 |
| Melanogenesis | 4916 | 101 | 32 | 11 | 2.172 |
| Glioma | 5214 | 65 | 28 | 11 | 2.165 |
| Adherens junction | 4520 | 73 | 28 | 11 | 2.145 |
| Bacterial invasion of epithelial cells | 5100 | 70 | 32 | 11 | 2.141 |
| Endocytosis | 4144 | 201 | 60 | 11 | 2.098 |
| Chemokine signaling pathway | 4062 | 189 | 47 | 11 | 2.05 |

**TABLE S4: KEGG Pathway Ranking Summary (PDS vs BSS, Score>2.0)**

| Term | Term ID | Total genes of the term | Union targets in the term | miRNAs in the term | Score |
| --- | --- | --- | --- | --- | --- |
| Axon guidance | 4360 | 129 | 55 | 10 | 4.139 |
| Pathways in cancer | 5200 | 325 | 105 | 11 | 3.998 |
| Erbb signaling pathway | 4012 | 87 | 42 | 11 | 3.114 |
| Focal adhesion | 4510 | 199 | 68 | 11 | 3.032 |
| Insulin signaling pathway | 4910 | 137 | 47 | 11 | 2.89 |
| Regulation of actin cytoskeleton | 4810 | 213 | 76 | 11 | 2.805 |
| TGF-beta signaling pathway | 4350 | 84 | 30 | 11 | 2.779 |
| Wnt signaling pathway | 4310 | 150 | 47 | 11 | 2.661 |
| MAPK signaling pathway | 4010 | 272 | 80 | 11 | 2.625 |
| Neurotrophin signaling pathway | 4722 | 127 | 47 | 11 | 2.454 |
| Calcium signaling pathway | 4020 | 177 | 44 | 11 | 2.447 |
| Chronic myeloid leukemia | 5220 | 73 | 33 | 11 | 2.368 |
| Gap junction | 4540 | 90 | 32 | 10 | 2.264 |
| Prostate cancer | 5215 | 89 | 33 | 11 | 2.155 |
| Gnrh signaling pathway | 4912 | 101 | 32 | 11 | 2.095 |
| Endocytosis | 4144 | 201 | 60 | 11 | 2.053 |
| Melanogenesis | 4916 | 101 | 32 | 11 | 2.047 |
| Bacterial invasion of epithelial cells | 5100 | 70 | 32 | 11 | 2.045 |
| Gastric acid secretion | 4971 | 74 | 25 | 11 | 2.026 |
| Glioma | 5214 | 65 | 28 | 11 | 2.02 |

**TABLE S5: GO Molecular function Ranking Summary (PDS vs HV, Score>2.0)**

| Term | Term ID | Total genes of the term | Union targets in the term | miRNAs in the term | Score |
| --- | --- | --- | --- | --- | --- |
| Transcription activator activity | GO:0016563 | 300 | 102 | 11 | 4.921 |
| Transcription factor binding transcription factor activity | GO:0000989 | 368 | 108 | 11 | 4.35 |
| Transcription repressor activity | GO:0016564 | 285 | 94 | 11 | 4.16 |
| RNA polymerase II transcription factor activity | GO:0003702 | 264 | 78 | 11 | 3.754 |
| Lipid binding | GO:0008289 | 381 | 85 | 10 | 2.103 |
| Chromatin binding | GO:0003682 | 189 | 54 | 11 | 2.071 |

**TABLE S6: GO Molecular function Ranking Summary (PDS vs BSS, Score>2.0)**

| Term | Term ID | Total genes of the term | Union targets in the term | miRNAs in the term | Score |
| --- | --- | --- | --- | --- | --- |
| Transcription activator activity | GO:0016563 | 300 | 102 | 11 | 4.674 |
| Transcription factor binding transcription factor activity | GO:0000989 | 368 | 108 | 11 | 4.581 |
| Transcription repressor activity | GO:0016564 | 285 | 94 | 11 | 3.982 |
| RNA polymerase II transcription factor activity | GO:0003702 | 264 | 78 | 11 | 3.812 |
| Chromatin binding | GO:0003682 | 189 | 54 | 11 | 2.205 |
